# Potential cost-savings from the use of the biosimilars filgrastim, infliximab and insulin glargine in Canada: a retrospective analysis

**DOI:** 10.1186/s12913-019-4680-2

**Published:** 2019-11-12

**Authors:** Kerry Mansell, Hishaam Bhimji, Dean Eurich, Holly Mansell

**Affiliations:** 1 College of Pharmacy and Nutrition, University of Saskatchewan, Saskatoon, SK S7N 2Z4 Canada; 2 School of Public Health, University of Alberta, Edmonton, AB T6G 2E1 Canada

**Keywords:** Biosimilars, Cost-savings, Filgrastim, Infliximab, Insulin glargine

## Abstract

**Background:**

In 2014 and 2015, biosimilars for the drugs filgrastim, infliximab, and insulin glargine were approved for use in Canada. The introduction of biosimilars in Canada could provide significant cost savings for the Canadian healthcare system over originator biologic drugs, however it is known that the use of biosimilars varies widely across the world. The aim of this study was to estimate the use of biosimilars in Canada and potential cost-savings from their use.

**Methods:**

We performed a retrospective analysis of Canadian drug purchases for filgrastim, infliximab, and insulin glargine from July 2016 to June 2018. This was a cross-sectional study and the time horizon was limited to the study period. As a result, no discounting of effects over time was included. Canadian drugstore and hospital purchases data, obtained from IQVIA™, were used to estimate the costs per unit and unit volume for biosimilars and originator biologic drugs within each province. Potential cost-savings were calculated as a product of the units of reference originator product purchased and the cost difference between the originator biologic and its corresponding biosimilar.

**Results:**

The purchase of biosimilars varied by each province in Canada, ranging from a low of 0.1% to a high of 81.6% of purchases. In total, $1,048,663,876 Canadian dollars in savings could have been realized with 100% use of biosimilars over the originator products during this 2 year time period. The potential savings are highest in the province of Ontario ($349 million); however, even in smaller markets (PEI and Newfoundland), $28 million could have potentially been saved. Infliximab accounted for the vast majority of the potential cost-savings, whereas the purchases of the biosimilar filgrastim outpaced that of the originator drug in some provinces. In sensitivity analyses assuming only 80% of originator units would be eligible for use as a biosimilar, $838 million dollars in cost savings over this two-year time period would still have been realized.

**Conclusions:**

The overall use of biosimilar drugs in Canada is low. Policy makers, healthcare providers, and patients need to be informed of potential savings by increased use of biosimilars, particularly in an increasingly costly healthcare system.

## Background

Biologic drugs first became available in Canada with the introduction of rDNA insulin in 1983 [1]. Since then, dozens of biologic drugs have been introduced in Canada, and in 2017 biologics represented 7 of the top 10 selling patented medications [2]. This is due, in part, to their significant advancements in the treatment of certain chronic and life-threatening conditions. Although biologic drugs are now common treatments for several disease states, their increased use has had a significant financial impact on private and public drug plans. Overall, Canadian spending on biologic drugs increased from $0.8 billion in 2006 to $3.6 billion in 2016, accounting for 15.9% of pharmaceutical sales for the entire country [3]. Further, these costs may underestimate the total cost of biologics, as many must be administered parenterally in a hospital setting, which requires additional resources beyond drug costs alone.

Recently, Health Canada has introduced a regulatory framework that allows for biosimilar drugs to be sold in Canada. Biosimilars are not generic medications; they are considered similar but not identical to the originator drug, due to the complexity of biologic molecules and their manufacturing process [4]. For biosimilars to be approved for use, stringent requirements including human clinical trials must be fulfilled. Once similarity has been established from structural and functional studies, some inferences can be made about the safety and efficacy of a biosimilar product. For example, though safety and efficacy must still be demonstrated in human clinical trials, studies might not be carried out for all labeled indications [4]. By comparison, generic drugs do not need to demonstrate safety and efficacy for market approval; rather, pharmacokinetic studies are sufficient to demonstrate relative bioequivalence to the reference product [5]. Despite some key differences, biosimilars and generics both serve a common economic function by offering comparable performance to the originator drug at a discounted price.

In 2014 and 2015, biosimilars for three widely-used medications became available for sale in Canada: infliximab, insulin glargine, and filgrastim [6]. Due to their common use and the significant cost associated with the originator product, the introduction of biosimilars for these biologic drugs could represent significant savings for the Canadian healthcare system and patients. The aim of this study was to examine national spending on these drugs in order to estimate potential cost-savings from the use of biosimilars over their respective originator product.

## Methods

This study was a retrospective analysis of Canadian drug purchases of the drugs infliximab (Remicade®, Inflectra®), insulin glargine (Lantus®, Basaglar®), and filgrastim (Neupogen®, Grastofil®), between July 2016 and June 2018. The three biosimilars evaluated are Inflectra®, Basaglar®, and Grastofil®, and the three originator biologics are Remicade®, Lantus® and Neupogen®. This was a cross-sectional study and the time horizon was limited to the year of study. As a result, no discounting of effects over time was included. The data used to estimate drug purchases and potential cost savings were obtained from IQVIA™, a multi-national healthcare analytics company [7]. IQVIA™ performed a Canadian Drugstore and Hospital Purchases (CDH) audit to estimate the dollar value and unit volume of Canadian pharmaceutical purchases by the major types of outlets within the retail and hospital sectors. From the CDH collective, a sample of outlets is selected according to IQVIA sampling methodology, which is proprietary and unavailable to the researchers. The projection methodology is then applied to create total national and regional/provincial estimates of pharmaceutical sales by manufacturer.

At the time of data collection, the CDH panel consisted of over 2800 drugstore outlets and 680 hospital sector type outlets. Sampling for this audit covers greater than one-third of the retail market and 88% of the hospital market. The drugstore sample and market are stratified by size (small, medium, large) and region (British Columbia, Alberta, Saskatchewan, Manitoba, Ontario, Quebec, Nova Scotia, New Brunswick and Newfoundland/Prince Edward Island). The hospital sample and market are stratified by region and type (general, long-term care, pediatric, psychiatric, cancer centers, government and all other specialties). General hospitals and long-term care centers are further stratified by size (small vs. large).

CDH data were provided for each specific dosage form and strength for all six of the drugs studied. For each drug, the total amount of dollars spent and unique units billed were provided for every province on a month-by-month basis. The data were collapsed into quarterly time periods for reporting. These data were provided as two separate data sets: one for drugstore dollars and drugstore units, and one for hospital dollars and hospital units. The price per drug unit was then calculated by dividing the total dollars spent on purchasing by the number of units purchased for each unique product for every province. Where there was an originator product available on the market but no comparative biosimilar dose or dosage form, these data were excluded and we assumed that no cost savings would be made.

In our base case analysis, for each unique original comparator product, potential cost-savings were calculated as a product between the units of reference originator product purchased and the cost difference between the originator product and its biosimilar on a quarterly basis for each province. The base case analysis assumed all originator units were expected to be made available for conversion to a biosimilar. In practice, this may not always be the case, as some patients may not tolerate the biosimilars or have a difference in response. As a result, in a sensitivity analysis we assumed 20% of all units would not be eligible to be converted to a biosimilar and recalculated the total cost savings, based on previous data and a conservative estimate [8].

## Results

The total units and the amount of dollars spent on purchases between July 1st, 2016 and June 30th, 2018 for both originator and biosimilar products for infliximab, filgrastim, and insulin glargine as per the CDH audit are described in Table 1. With respect to the biosimilars during this time period, Basaglar® accounted for 7.8% (619,155/7,947,223 units) of all insulin glargine purchases, Grastofil® accounted for 27.0% (382,254/1,415,762 units) of filgrastim purchases, and Inflectra® accounted for 3.0% (67,330/2,257,797 units) of infliximab purchases in the CDH audit. Overall, the three biosimilar drugs accounted for 4.2% ($108,666,140/$2,646,773,824) of total drug dollar purchases while the originator products accounted for 95.8% ($2,538,107,684/$2,646,773,824) of total drug dollar purchases.

**Table 1:** Description of Canadian Drugstore and Hospital (CDH) purchases and potential dollar savings from July 2016 to June 2018 for all provinces

	CDH Purchases	Units Purchased	Price Per Unit	Average Discounted Price (%)	Average Realized Savings through use of biosimilar	Unrealized Savings	Overall savings realized (%)
Lantus®	$ 141,135,286	7,328,068	$ 19.26	$ 4.07 (21.1%)	$ 2,519,961	$29,825,236	7.8%
Basaglar®	$ 9,402,061	619,155	$ 15.19				
Neupogen®	$ 204,152,590	1,033,508	$ 197.53	$ 35.17 (17.8%)	$ 13,443,873	$36,348,476	27.0%
Grastofil®	$ 62,061,576	382,254	$ 162.36				
Remicade®	$ 2,192,819,808	2,190,467	$ 1001.07	$ 448.53 (44.8%)	$ 30,199,524	$982,490,164	3.0%
Inflectra®	$ 37,202,503	67,330	$ 552.54				
TOTAL					$ 46,163,358	$1,048,663,876	4.2%

All dollar figures are in Canadian dollars

Estimates of realized and unrealized savings are based on the average discounted price amongst all provinces

Unrealized savings is calculated as the average price difference between the originator and biosimilar amongst all provinces, multiplied by the number of originator units sold

**Fig. 1:**
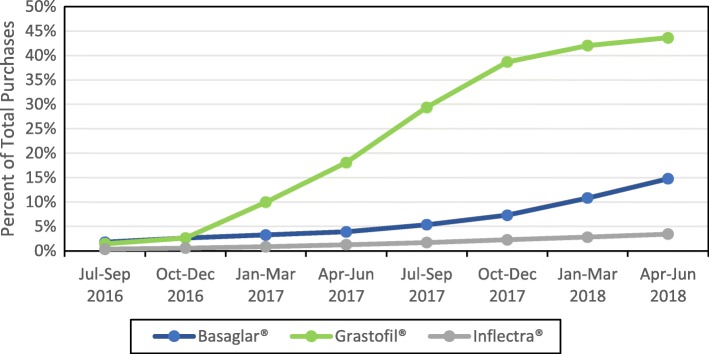
Quarterly purchases of the biosimilars Inflectra®, Basaglar®, and Grastofil®. Percent of Total Purchases represents the percent of time the biosimilars Basaglar®, Grastofil®, and Inflectra® were purchased when considering all purchases for both the biosimilar (Basaglar®, Grastofil®, and Inflectra®) and originator drugs (Lantus®, Neupogen®, and Remicade®)

Purchases of each of the three biosimilar drugs increased from the start of the study period to the end of the study period. Initially, during the time period of July to September 2016, the three biosimilars Inflectra®, Basaglar®, and Grastofil® accounted for 0.3% ($924,107/$265,066,516), 1.8% ($338,062/$19,084,914) and 1.5% ($496,114/$33,813,822) of total dollar purchases respectively. By the end of the study period, from April to June 2018, this dollar amount increased to 3.4% ($9,921,465/$289,844,413), 14.8% ($2,875,314/$19,458,498), and 43.6% ($15,130,096/$34,672,908) respectively (see Fig. 1).

**Fig. 2:**
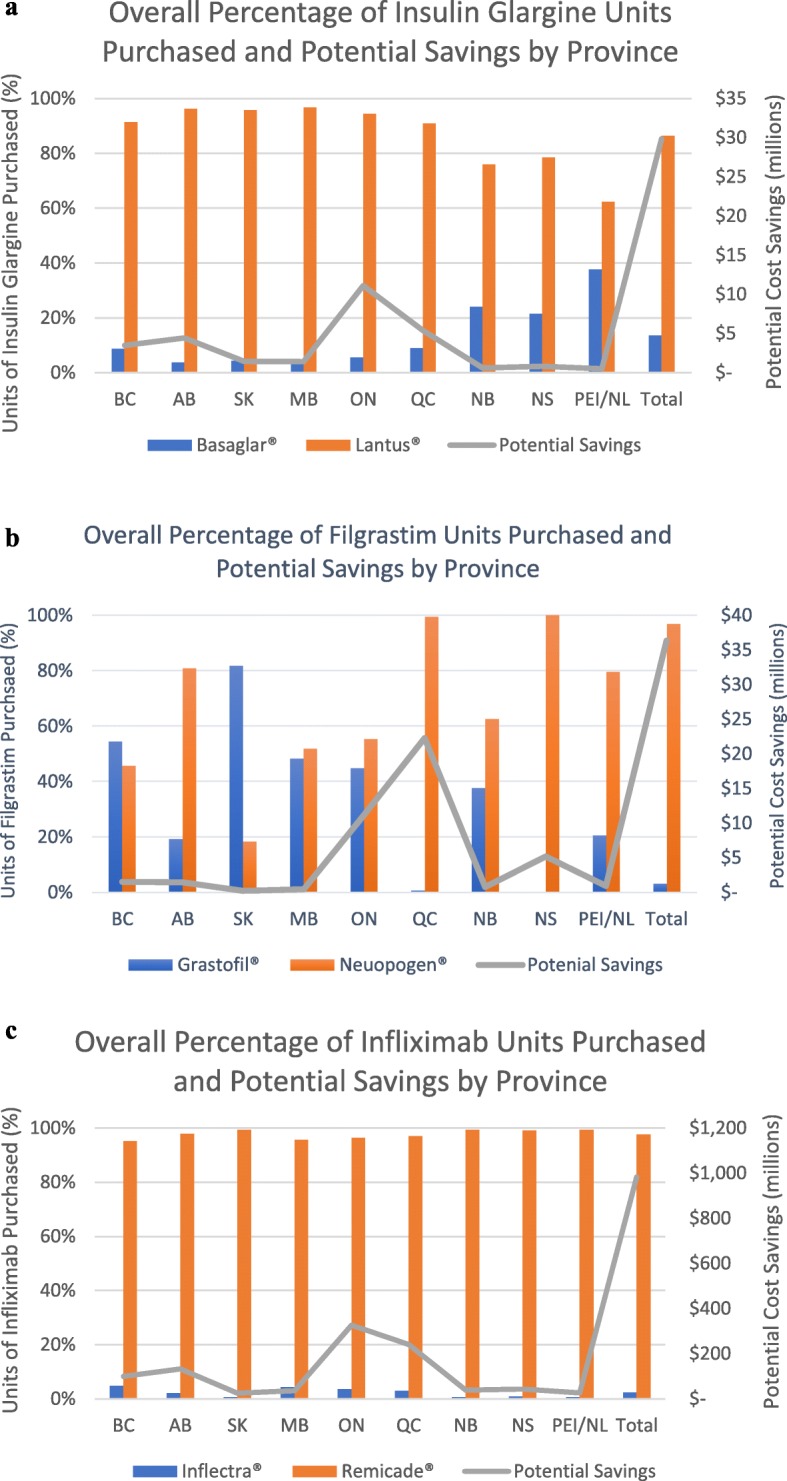
**a**. Overall units purchased of insulin glargine by province. All dollar figures are in Canadian dollars. BC=British Columbia, AB = Alberta, SK=Saskatchewan, MB = Manitoba, ON=Ontario, QC = Quebec, NB=New Brunswick, NS=Nova Scotia, PEI/NL = Prince Edward Island / Newfoundland. Potential Savings represents the potential savings that could have been realized if the biosimilar drug Basaglar® were purchased instead of the originator drug Lantus®. **b** Overall units purchased of filgrastim by province. All dollar figures are in Canadian dollars. BC=British Columbia, AB = Alberta, SK=Saskatchewan, MB = Manitoba, ON=Ontario, QC = Quebec, NB=New Brunswick, NS=Nova Scotia, PEI/NL = Prince Edward Island / Newfoundland. Potential Savings represents the potential savings that could have been realized if the biosimilar drug Grastofil® were purchased instead of the originator drug Neupogen®. **c** Overall units purchased of infliximab by province. All dollar figures are in Canadian dollars. BC=British Columbia, AB = Alberta, SK=Saskatchewan, MB = Manitoba, ON=Ontario, QC = Quebec, NB=New Brunswick, NS=Nova Scotia, PEI/NL = Prince Edward Island / Newfoundland. Potential Savings represents the potential savings that could have been realized if the biosimilar drug Inflectra® were purchased instead of the originator drug Remicade®

The use of biosimilar agents varied across the provinces (Fig. 2). The overall number of units purchased for the biosimilar Basaglar® ranged from a low of 3.2% (10,505/325,736) in Manitoba, to a high of 37.7% (63,698/169,199) in Prince Edward Island/Newfoundland. The number of units purchased for the biosimilar Grastofil® ranged from a low of 0.1% (22/28,494) in Nova Scotia, to a high of 81.6% (19,271/23,610) in Saskatchewan. The number of units purchased for the biosimilar Inflectra® ranged from a low of 0.6% (337/53,115) in Prince Edward Island/Newfoundland, to a high of 4.8% (10,787/224,258) in British Columbia. A detailed description of purchases by province are described in the Additional file 1, 2 and 3.

In the base case, an additional $1,048,663,876 could have been potentially saved nationally during this two-year time period if there had been 100% uptake of the biosimilars over the originator product, based on the average discounted price (Table 1). The savings would have been highest in the largest province of Ontario where an additional $349,443,270 could have been saved had there been 100% uptake of the biosimilars (Table 2). Even in the smallest region of Prince Edward Island and Newfoundland, an additional $28,220,107 could have potentially been saved with 100% use of the biosimilars (Table 2). Overall, infliximab accounts for the majority of the cost-savings, with potential to have saved approximately an additional one billion dollars on its own within this two-year period (Table 1). Even in sensitivity analyses where we assume only 80% of units would be eligible for conversion, the biosimilars would still have resulted in an additional $838 million dollars in cost savings over this two-year time period.

**Table 2:** Realized and Unrealized Savings for the biosimilars Basaglar®, Grastofil®, and Inflectra® Relative to Captured Market Share by Province

Combined Total		BC	AB	SK	MB	ON	QC	NB	NS	PEI/NL	Total
Relative Market Share	25%	$27,928,392	$35,351,847	$7,050,947	$10,825,836	$92,858,064	$69,340,492	$10,224,942	$12,572,878	$7,232,388	$273,720,060
	50%	$55,856,785	$70,703,694	$14,101,894	$21,651,672	$185,716,128	$138,680,983	$20,449,885	$25,145,756	$14,464,775	$547,440,121
	75%	$83,785,177	$106,055,541	$21,152,840	$32,477,508	$278,574,191	$208,021,475	$30,674,827	$37,718,634	$21,697,163	$821,160,181
	100%	$111,713,570	$141,407,387	$28,203,787	$43,303,343	$371,432,255	$277,361,966	$40,899,770	$50,291,511	$28,929,550	$1,094,880,242
Realized Savings	($)	$7,174,406	$3,332,450	$1,174,422	$2,232,171	$21,988,985	$8,019,828	$912,786	$615,111	$709,443	$46,168,848
	(%)	6.42%	2.36%	4.16%	5.15%	5.92%	2.89%	2.23%	1.22%	2.45%	4.22%
Unrealized Savings	($)	$104,539,164	$138,074,937	$27,029,365	$41,071,172	$349,443,270	$269,342,139	$39,986,984	$49,676,400	$28,220,107	$1,048,711,394
	(%)	93.58%	97.64%	95.84%	94.85%	94.08%	97.11%	97.77%	98.78%	97.55%	95.78%

All dollar figures are in Canadian dollars

*BC* British Columbia, *AB* Alberta, *SK* Saskatchewan, *MB* Manitoba, *ON* Ontario, *QC* Quebec, *NB* New Brunswick, *NS* Nova Scotia, *PEI/NL* Prince Edward Island / Newfoundland

Realized savings is calculated as the difference in price between the originator and biosimilar in each particular province, multiplied by the number of biosimilar units sold

Unrealized savings is calculated as the difference in price between the originator and biosimilar in each particular province, multiplied by the number of originator units sold

## Discussion

From July 1st 2016 until June 30th, 2018, approximately one billion dollars in savings could have been realized through exclusive purchasing of biosimilar drugs for infliximab, filgrastim, and insulin glargine as opposed to the originator products in Canada. Overall, approximately $46 million dollars was saved through purchases of the biosimilar drugs, which accounts for only 4.2% of potential total savings. There was a trend in the data towards greater use of biosimilars over time; however, overall use remained relatively low. Substantial differences between the three products and individual provinces were noted; the highest percent of biosimilar use occurred with Grastofil**®** in Saskatchewan (81.6%), as opposed to some provinces purchasing the biosimilars Inflectra**®** and Grastofil**®** less than 1% of the time. In Canada, each province is responsible for their own spending on health care services as opposed to one national health care plan, which is one reason why there are differences observed between the provinces.

The slow uptake of biosimilar drugs is not unique to Canada. A study by Grabowski et al. reported that Sweden and Germany observed fast uptake of a filgrastim biosimilar, while Italy, France, and the United Kingdom lagged behind [9]. Uptake can also be highly variable even within a single healthcare system, as one study observed that 90% of inpatients in eastern Massachusetts used the biosimilar tbo-filgrastim, compared to only 50% of outpatients [10]. It is unsurprising that biosimilars have not been able to penetrate the Canadian market as generic drugs do, given their complexity, lack of interchangeability, and lower discounting than in other countries [3]. Lack of patient awareness, knowledge about biosimilars, and discomfort from prescribers further explain their slow uptake [11, 12]. Apprehension with biosimilar use generally pertains more to switching therapies for stable patients as opposed to initiation in treatment-naïve patients [13]; however, several studies have found that switching between agents does not confer any additional risk or lead to poorer clinical outcomes [13–15]. Although the safety and efficacy profile of biosimilar drugs cannot be assured to be 100% due to their complexity, existing data reassure that is very likely that biosimilar and originator biologics are relatively equal as it pertains to both [16–19].

Potential cost savings with biosimilars has been studied before, primarily with infliximab in European countries. When the biosimilar infliximab first became available in 2014 in Norway, it captured less than 10% of the market share, whereas now it has captured greater than 90% [20]. Similar market share has been observed in other Nordic countries [20, 21]. Some factors attributed to the large uptake in certain countries include a lower price, setting, competition through tendering, and recommendations from key opinion leaders [20, 21]. Depending on the degree of discounting, uptake, setting, and indications being studied, studies projected infliximab to save anywhere from 2 to 493 million Euros [22–28]. The Patented Medicine Prices Review Board (PMPRB) of Canada reported that if the use of the biosimilar infliximab had been the same as the Organization for Economic Co-operation and Development (OECD) median in 2015, it would have saved $41.7 million in drug expenditures [3]. The PMPRB further predicted that with a high-uptake, high-discount scenario, the use of the biosimilars infliximab, filgrastim and insulin glargine could amount to $514 million, $62 million, and $130 million in savings, respectively, in 2019 [3]. Based on our 2018 data, it is very unlikely these savings will be realized without a fundamental shift in the use of biosimilars in Canada.

This study has several limitations. Firstly, there is another biosimilar for infliximab which was not included in this analysis; however, it is unlikely it would have had a significant effect given the observed trends. Further, the CDH data are drawn from intake drug purchase and do not account for the outflow of drugs sold from retail or hospital outlets. Although this may overestimate the total cost savings projected, the same relative cost saving would have been expected even if the total number of units actually dispensed was lower. Although the data are from a representative sample of retail and hospital outlets across Canada, not all outlets are included. Additionally, in order to calculate potential unit cost-savings, the publicly-available drug prices were used, which may not reflect the actual cost of these medications once private purchase agreements are accounted for. Hence, it is likely that our potential cost-savings calculations may be overestimated, although the savings would still be expected to be substantial. For example, even with a 50% reduction in the cost differences with a private purchase agreement in place, approximately half a billion dollars could still have been saved. Finally, for comparisons we assumed that the safety and efficacy of the originator and biosimilar drugs were similar. Although reasonable, due to the complexity of the drugs in question, differences in safety or efficacy could exist which were not accounted for in the models.

## Conclusions

Overall, the amount of cost savings that could be realized through increased use of the biosimilars filgrastim, infliximab, and insulin glargine is significant. Given the relatively low use observed in Canada with biosimilars as opposed to almost universal use in some European countries, important discussions by Canadian stakeholders need to occur as soon as possible. This will become increasingly important as increasing amounts of biosimilars become approved for use. Without new policies prioritizing the use of biosimilars, it is unlikely major costs savings to patients and public and private insurance plans will be realized in the near future.

## Supplementary information

**Additional file 1.** Realized and Unrealized Savings for Inflectra® Relative to Captured Market Share by Province.

**Additional file 2.** Realized and Unrealized Savings for Basaglar® Relative to Captured Market Share by Province.

**Additional file 3.** Realized and Unrealized Savings for Grastofil® Relative to Captured Market Share by Province.

## Data Availability

The data that support the findings of this study are available from IQVIA™ but restrictions apply to the availability of these data, which were used under license for the current study, and so are not publicly available.

## Abbreviations

AB: Alberta
BC: British Columbia
CDH: Canadian Drugstore and Hospital
MB: Manitoba
NB: New Brunswick
NL: Newfoundland
NS: Nova Scotia
OECD: Organization for Economic Co-operation and Development
ON: Ontario
PEI: Prince Edward Island
PMPRB: Patented Medicine Prices Review Board
QC: Quebec
rDNA: Recombinant Deoxyribonucleic Acid
SK: Saskatchewan
