# Correction to: Potential cost-savings from the use of the biosimilars filgrastim, infliximab and insulin glargine in Canada: a retrospective analysis

**DOI:** 10.1186/s12913-019-4829-z

**Published:** 2019-12-18

**Authors:** Kerry Mansell, Hishaam Bhimji, Dean Eurich, Holly Mansell

**Affiliations:** 1 College of Pharmacy and Nutrition, University of Saskatchewan, Saskatoon, SK S7N 2Z4 Canada; 2 School of Public Health, University of Alberta, Edmonton, AB T6G 2E1 Canada

**Correction to: BMC Health Serv Res**

**https://doi.org/10.1186/s12913-019-4680-2**

In the original publication of this article [1], there is a mistake in the Fig. 2a, b, and c. The mistake is in the very last column where it shows TOTAL. The updated Fig. 2 is shown below. Additionally, a footnote “Total is based on the average discounted price amongst all provinces.” should be added to Table 2 and Additional files 1, 2, and 3.

**Fig. 2:**
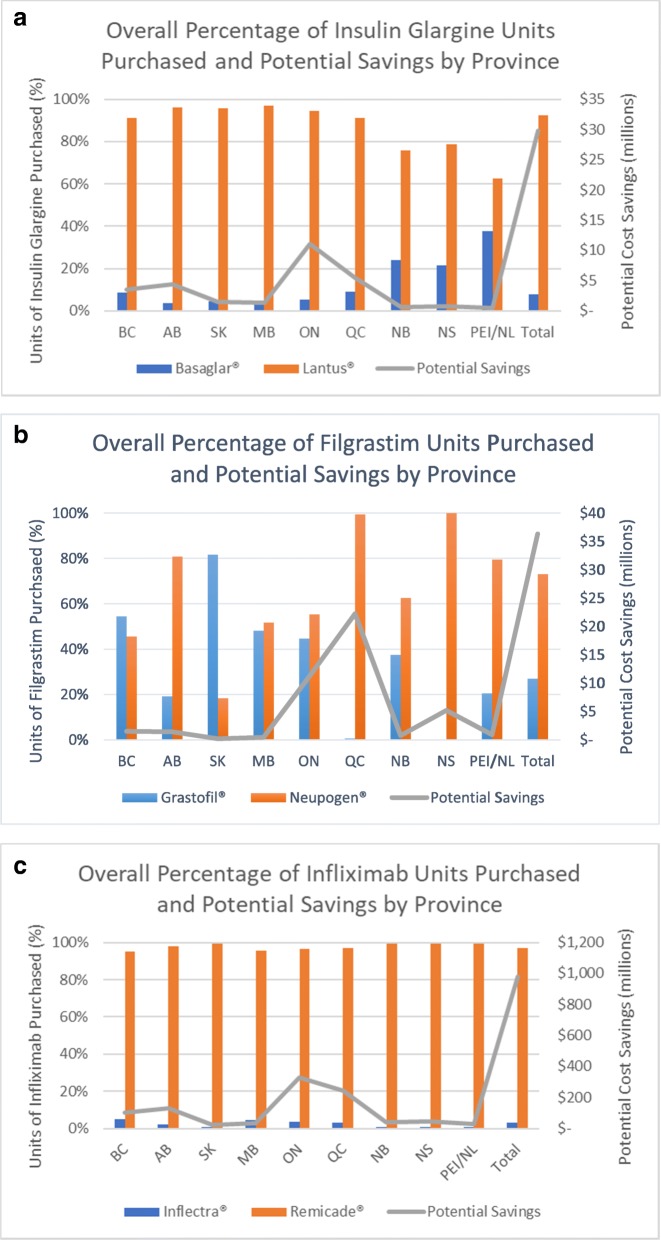
**a**. Overall units purchased of insulin glargine by province. All dollar figures are in Canadian dollars. BC=British Columbia, AB = Alberta, SK=Saskatchewan, MB = Manitoba, ON=Ontario, QC = Quebec, NB=New Brunswick, NS=Nova Scotia, PEI/NL = Prince Edward Island / Newfoundland. Potential Savings represents the potential savings that could have been realized if the biosimilar drug Basaglar® were purchased instead of the originator drug Lantus®. **b** Overall units purchased of filgrastim by province. All dollar figures are in Canadian dollars. BC=British Columbia, AB = Alberta, SK=Saskatchewan, MB = Manitoba, ON=Ontario, QC = Quebec, NB=New Brunswick, NS=Nova Scotia, PEI/NL = Prince Edward Island / Newfoundland. Potential Savings represents the potential savings that could have been realized if the biosimilar drug Grastofil® were purchased instead of the originator drug Neupogen®. **c** Overall units purchased of infliximab by province. All dollar figures are in Canadian dollars. BC=British Columbia, AB = Alberta, SK=Saskatchewan, MB = Manitoba, ON=Ontario, QC = Quebec, NB=New Brunswick, NS=Nova Scotia, PEI/NL = Prince Edward Island / Newfoundland. Potential Savings represents the potential savings that could have been realized if the biosimilar drug Inflectra® were purchased instead of the originator drug Remicade®

**Table 2:** Realized and Unrealized Savings for the biosimilars Basaglar®, Grastofil®, and Inflectra® Relative to Captured Market Share by Province

Combined Total		BC	AB	SK	MB	ON	QC	NB	NS	PEI/NL	Total
Relative Market Share	25%	$27,928,392	$35,351,847	$7,050,947	$10,825,836	$92,858,064	$69,340,492	$10,224,942	$12,572,878	$7,232,388	$273,720,060
	50%	$55,856,785	$70,703,694	$14,101,894	$21,651,672	$185,716,128	$138,680,983	$20,449,885	$25,145,756	$14,464,775	$547,440,121
	75%	$83,785,177	$106,055,541	$21,152,840	$32,477,508	$278,574,191	$208,021,475	$30,674,827	$37,718,634	$21,697,163	$821,160,181
	100%	$111,713,570	$141,407,387	$28,203,787	$43,303,343	$371,432,255	$277,361,966	$40,899,770	$50,291,511	$28,929,550	$1,094,880,242
Realized Savings	($)	$7,174,406	$3,332,450	$1,174,422	$2,232,171	$21,988,985	$8,019,828	$912,786	$615,111	$709,443	$46,168,848
	(%)	6.42%	2.36%	4.16%	5.15%	5.92%	2.89%	2.23%	1.22%	2.45%	4.22%
Unrealized Savings	($)	$104,539,164	$138,074,937	$27,029,365	$41,071,172	$349,443,270	$269,342,139	$39,986,984	$49,676,400	$28,220,107	$1,048,711,394
	(%)	93.58%	97.64%	95.84%	94.85%	94.08%	97.11%	97.77%	98.78%	97.55%	95.78%

All dollar figures are in Canadian dollars

*BC* British Columbia, *AB* Alberta, *SK* Saskatchewan, *MB* Manitoba, *ON* Ontario, *QC* Quebec, *NB* New Brunswick, *NS* Nova Scotia, *PEI/NL* Prince Edward Island / Newfoundland

Realized savings is calculated as the difference in price between the originator and biosimilar in each particular province, multiplied by the number of biosimilar units sold

Unrealized savings is calculated as the difference in price between the originator and biosimilar in each particular province, multiplied by the number of originator units sold

Total is based on the average discounted price amongst all provinces
