# Supplementary material for: Potential cost-savings from the use of the biosimilars filgrastim, infliximab and insulin glargine in Canada: a retrospective analysis
Source: BMC Health Serv Res. 2019 Nov 12;19:827. doi: 10.1186/s12913-019-4680-2 (PMC6852752; doi:10.1186/s12913-019-4680-2)
Supplement: Supplementary file 3 — Additional file 3. Realized and Unrealized Savings for Grastofil® Relative to Captured Market Share by Province. [file 12913_2019_4680_MOESM3_ESM.docx]

**Appendix 3: Realized and Unrealized Savings for Grastofil^®^ Relative to Captured Market Share by Province**

| **Grastofil^®^** | | **BC** | **AB** | **SK** | **MB** | **ON** | **QC** | **NB** | **NS** | **PEI/NL** | **Total** |
| --- | --- | --- | --- | --- | --- | --- | --- | --- | --- | --- | --- |
| Relative Market Share | 25% | $833,326 | $439,055 | $285,726 | $212,279 | $5,057,355 | $5,599,881 | $303,534 | $1,297,406 | $284,857 | $12,450,469 |
|  | 50% | $1,666,652 | $878,109 | $571,453 | $424,557 | $10,114,711 | $11,199,762 | $607,068 | $2,594,811 | $569,714 | $24,900,938 |
|  | 75% | $2,499,977 | $1,317,164 | $857,179 | $636,836 | $15,172,066 | $16,799,644 | $910,602 | $3,892,217 | $854,571 | $37,351,407 |
|  | 100% | $3,333,303 | $1,756,219 | $1,142,905 | $849,115 | $20,229,421 | $22,399,525 | $1,214,136 | $5,189,623 | $1,139,428 | $49,801,876 |
| Realized Savings | ($) | $1,812,449 | $337,449 | $932,864 | $409,383 | $9,047,489 | $137,664 | $455,158 | $4,007 | $233,959 | $13,446,445 |
|  | (%) | 54.37% | 19.21% | 81.62% | 48.21% | 44.72% | 0.61% | 37.49% | 0.08% | 20.53% | 27.00% |
| Unrealized Savings | ($) | $1,520,854 | $1,418,770 | $210,041 | $439,732 | $11,181,932 | $22,261,861 | $758,978 | $5,185,616 | $905,468 | $36,355,431 |
|  | (%) | 45.63% | 80.79% | 18.38% | 51.79% | 55.28% | 99.39% | 62.51% | 99.92% | 79.47% | 73.00% |

All dollar figures are in Canadian dollars

BC=British Columbia, AB=Alberta, SK=Saskatchewan, MB=Manitoba, ON=Ontario, QC=Quebec, NB= New Brunswick, NS=Nova Scotia, PEI/NL=Prince Edward Island / Newfoundland

Realized savings is calculated as the difference in price between the originator and biosimilar in each particular province, multiplied by the number of biosimilar units sold

Unrealized savings is calculated as the difference in price between the originator and biosimilar in each particular province, multiplied by the number of originator units sold
